# “I lock myself in my room and cry in frustration”: an analysis of adolescent behaviors of child-to-parent violence

**DOI:** 10.3389/fpsyt.2025.1524413

**Published:** 2025-02-03

**Authors:** Shirley Arias-Rivera, Barbara Lorence, Jesus Maya

**Affiliations:** ^1^ Departamento de Psicología, Universidad Loyola Andalucía, Seville, Spain; ^2^ Departamento de Psicología Evolutiva y de la Educación, Sevilla University, Seville, Spain; ^3^ Research Group HUM604 ‘Lifestyle Development in the Life Cycle and Health Promotion’, University of Huelva, Huelva, Spain

**Keywords:** child-to parent violence, adolescent behavior, emotional response, systematic analysis, intervention strategies

## Abstract

Child-to-parent violence (CPV) encompasses behaviors such as hitting, insulting, or threatening parents. Over the past decade, the number of CPV cases has increased significantly. While previous research has largely focused on classifying CPV behaviors and examining their causes, there is limited investigation into what happens immediately after CPV episodes. This study aims to describe the behaviors of adolescents following violent actions in both mild and severe cases of CPV. A randomized sample of 1,067 Spanish adolescents, participating in a national CPV project, was studied using the Child-to-Parent Aggression Questionnaire. Among them, 410 adolescents (41.91% boys and 57.84% girls) reported engaging in some form of CPV behavior in the past year, and 47 adolescents met the criteria for severe and repeated CPV. Specifically, 26 adolescents exhibited psychological and 27 exhibited physical CPV toward their mother, while 21 adolescents exhibited psychological and 15 physical CPV toward their father. Adolescents were asked, “What do you do after?” Following thematic analysis, adolescents’ responses were categorized into 6 themes and 17 sub-themes in mild cases. A possible sequence in adolescents’ responses was identified, divided into three phases. In the first phase, adolescents felt bad after their violent behavior, expressed remorse, reflected on it, and sought a safe place. In the second phase, apologizing to parents was the most common response, reported by 70.52% of adolescents. Finally, in the third phase, in addition to apologizing, adolescents attempted to talk with their parents, gave them a hug, or committed to not repeating the behavior. In contrast, 8% of adolescents normalized their behavior, joked about it, or justified their actions. In severe cases, most subthemes were consistent with those observed in mild CPV cases. However, in contrast to milder cases, severe cases showed a lower percentage of adolescents who felt bad or apologized and a higher proportion who normalized, avoided talking about, or justified their violent actions (23.4% of adolescents with severe CPV). This study highlights practical implications for interventions, such as the importance of helping them express their emotions, recognize the harm caused, identify safe spaces, people, or activities, and support them in the process of apologizing.

## 1 Introduction

Child-to-parent violence (CPV) is defined as a set of aggressive behaviors directed toward parents or caregivers. These behaviors may include physical aggression, insults, threats, and economic violence and are characterized by being recurrent, deliberate, and intentional (1). CPV has serious negative repercussions in both personal and family domains, as it deteriorates the emotional attachment between parents and children while disrupting family dynamics, creating an atmosphere of tension (2, 3). Over the past decade, the reported number of CPV cases has increased significantly (4). However, estimating the prevalence of CPV is difficult because many times no reports are made (5). Community-based studies in the U.S., Canada, the United Kingdom, and Spain indicate that severe physical violence is reported in 3%-5% of cases, while psychological aggression observed in 14.2% of cases in studies from the UK and Spain (6). Special attention should also be paid to the relationship between CPV and gender. According to various studies, observable differences exist in the type of violence exerted, the gender of parents and children, and the sample’s origin. Community-based investigations report higher levels of verbal (7) and psychological abuse perpetrated by daughters compared to sons (8), primarily directed toward mothers (9, 10). Studies with judicial samples have indicated that daughters exhibit greater controlling and dominating behaviors, as well as physical violence towards mothers, than sons (11). Additionally, mothers are significantly more likely to experience aggression from both sons and daughters (9, 10, 12).

Theoretical frameworks designed to understand violent behavior in general have been applied to analyze the causes of CPV. Key among these are the Intergenerational Theory of Violence (13) Social Information Processing Model (14), and Social Learning Theory (15), with a focus on aggressive cognitions—such as scripts, schemas, attitudes, and beliefs—as mechanisms of control (16). Additionally, factors that either promote or inhibit aggression are also considered (17). They have also been developed specific models for understanding CPV, including the Nested Ecological Model (18), the CPV Cycle (19), the Symptomatic Cycle (20), the model describing the loss of parental authority and the progressive increase in CPV (21), an integrative framework combining social control theory, differential association theory, and strain theory (22), a process model analyzing stressor, moderator, and mediator variables in CPV development (23), and the Negative Interaction Cycle (24). It is clear that this is a complex phenomenon which is explained through an interaction of personal, family and contextual factors (6, 25).

CPV is recognized as a relational problem in which violence becomes an entrenched means of interaction within the family system (18, 19, 26), which has been learned and reinforced over time (19, 27). From the perspective of adolescents, the reasons that lead them to engage in this type of violence have been classified as reactive, proactive and affective (28). Among the variables analyzed those of an emotional (29) and socio-cognitive nature have aroused particular interest (30), has been linked to difficulties in self-control, low tolerance for frustration, lack of empathy and emotional dysregulation (31, 32), anger, a tendency to interpret parents’ actions or intentions in a negative or aggressive manner (hostile attribution), and the belief that aggression can produce positive results, such as avoiding tasks or gaining privileges (17, 33, 34). From a systemic perspective, the identified variables primarily pertain to challenges in parenting skills (35, 36), with a particular emphasis on bidirectional patterns of submission-hostility or hostility-hostility associated with violent acts (28, 37), family communication dynamics (6), and the history of adolescents’ exposure to parental domestic violence and/or child maltreatment (4, 6, 11, 38). Typically, conflict emerges as a response to parental rule-setting or unmet demands from the child. When parents deny these requests, the child may respond violently, leading parents to adopt a conciliatory stance to alleviate the tension and stress triggered by the situation (19). However, this conciliatory response is ultimately ineffective, as adolescents often interpret parental concessions as signs of submission, further intensifying their demands, aggression, and control strategies. Consequently, parents may either escalate their hostility or fully yield to the adolescent’s demands, reinforcing a coercive cycle that perpetuates the cycle of violence within the family (7, 19).

Omer (37) conceptualizes this parental response within two escalation frameworks: complementary and symmetrical escalation. Complementary escalation describes instances where parental submission amplifies the child’s demands, often involving dynamics of emotional blackmail, with parents acquiescing to avoid potential threats. Symmetrical escalation, conversely, occurs as mutual hostility increases, with each party perceiving themselves as the victim acting in self-defense. According to the theory of stress, CPV is a reactive and adaptive response to family aggression experienced by children within the home. Like the dynamics observed in partner violence, Roperti (24) describes the progression of CPV as an inverted pyramid. The stress accumulated from confronting parental figures causes violent and uncontrolled outbursts in adolescents, often followed by remorse due to emotional release. Parents do not know how to react by increasing tension in their relationships, which becomes a precursor of subsequent violent episodes, restoring the cycle of violence (8). On the other hand, the contextual variables studied highlight the influence of adolescent peer connections with individuals who engage in violent behavior, further reinforcing their actions (6, 34). Additionally, challenges parents face in accessing social support resources (35), and difficulties in school adaptation and learning (6), are significant factors.

Additionally, there is a significant body of knowledge on how individuals respond to and regulate themselves in the face of aversive or challenging events. Research has extensively examined coping strategies (39) as well as the adaptive and maladaptive regulation of emotions (40). More recent studies highlight the crucial role of flexibility in emotional management and regulation (41). It is known that individuals’ responses to stressful events vary depending on the nature of the event and the regulatory strategies employed. These strategies are not intrinsically suitable or unsuitable; rather, their effectiveness depends on regulatory flexibility. Regulatory flexibility (41) involves the capacity to simultaneously evaluate the context, the repertoire of available regulatory strategies, and feedback on the effectiveness of the applied strategy, thereby allowing the strategy to be maintained, adjusted, or replaced as needed. Internal self-assessment plays a fundamental role in self-regulation; however, social feedback on the effectiveness of the chosen strategy is a decisive element in its development (41).

Another important aspect is the gender differences in the use of coping strategies. Various studies indicate that girls tend to seek social support and engage in cooperative conflict resolution, while boys are more likely to adopt avoidant coping or aggressive strategies to address violence (42–44). Additionally, regarding studies that explore gender differences among adolescents who perpetrate child-to-parent violence, findings suggest that girls tend to be more attentive to their own emotions and more empathetic than boys. However, girls often experience greater difficulties in regulating their emotions (45, 46).

Functional explanations of emotions in a social context, as proposed by Keltner and Haidt (47), emphasize that emotional expressions not only facilitate communication but also modulate others’ behavior. In this sense, it is suggested that the intensity of emotions generated during CPV acts surpasses the family system’s capacity to respond. This system, characterized by a limited repertoire of emotional regulation strategies, tends to produce repetitive and similar responses in both parents and adolescents, thereby reinforcing the cycle of violence. In this context, intervention programs are designed with objectives that include helping individuals recognize various types of emotions and understand their influence on human behavior, especially in instances of parental abuse (48).

With the aim of understanding the progression of violent behavior over time, CPV trajectories have been analyzed from the perspective of adolescent aggressors. While understanding the history of CPV over time can provide valuable insights for prevention (49), knowing what happens afterward is essential for intervention. Knowing the events that occur after CPV offers an important perspective, as it not only validates the right of these adolescents to directly narrate their experiences—thus giving them a voice in the process—but also helps tailor interventions to better address their specific needs and experiences. Ultimately, this approach provides a more comprehensive understanding of violence and its potential solutions (50).

Emotions experienced after committing violent acts, such as shame and guilt, can serve as catalysts in the rehabilitation process due to their roles in promoting altruistic behavior and inhibiting antisocial tendencies. Remorse is identified as a central component of the guilt experience. Guilt, understood as an internally and privately generated feeling of remorse, is more closely associated with moral transgressions (51). In line with this, Reintegrative Shaming Theory (RST, 41) proposes that feelings of shame following violent behaviors can facilitate the perpetrator’s apology. In this way, reflection on the violent act and the emotions generated in both the perpetrator and the victim can support the processes of apology and reparative behaviors, suggesting the inclusion of these components in intervention processes.

The current state of research on CPV has provided various insights into explanatory theories and variables associated with CPV. However, studies focused on identifying the key components for CPV programs and interventions remain necessary. Therefore, analyzing the specific moment when CPV occurs and what follows represents an innovative approach to this topic with potential practical implications for intervention. Additionally, research has predominantly been quantitative, highlighting the need for more qualitative studies that give a voice to both adolescents and parents (52). Understanding adolescents’ actions following CPV incidents can support the development of interventions by identifying key components as preventive strategies in mild cases and intervention strategies in severe cases. This study aims to describe the behaviors of adolescents following violent actions in both mild and severe cases of CPV.

## 2 Methods

### 2.1 Participants

The sample for this study (410 boys and girls) was selected from the adolescent participants (*n* = 1067, between the ages of 11 and 18) of the national project in Spain, “Child-to-parent violence in adolescence: detection, psychosocial profiles, and action strategies.” These confirmed that, at some point in the past year, they had performed acts of violence to some degree (*either physical or psychological*) toward one of their caregivers. They therefore responded to the supplementary question “What do you do after?”.

The 410 participants belonged to 17 high schools in Spain. The distribution of participants by region was as follows: 23.66% of Andalusia, 34.39% of Extremadura, 11.22% Community of Madrid, 23.90% Castilla-La Mancha y 6.83% Balearics Island. 41% (*n* = 168) of adolescents were schooled in rural areas (< 50000 habitants) while the rest (59%; *n* = 242) were in areas considered urban. According to gender, 171 were boys (41.91%), 236 were girls (57.84%) and three did not identify with being a boy or a girl. The average age of the sample was 14.88 (*SD* = 1.85), with 28% between 11 and 13 years, 47.13% between 14 and 16 years, and 24.87% between 17 and 18 years. 69.51% (*n* = 285) of the adolescents lived with both parents and 30.49% (*n* = 125) had either separated parents or absent parents (*death or no relationship with children*), this information was not provided by the rest of the sample.

### 2.2 Measures

The results of this paper are derived from data collected using the instrument the Child-to-Parent Aggression Questionnaire (CPAQ-R, 53), which consists of 18 parallel items concerning psychological violence (e.g., “*You’ve threatened to hit him/her, even though you didn’t actually do it*”), physical violence (e.g., “*You’ve hit him/her with something that could hurt*”), directed at the father (9 items) and the mother (9 items) over the past year. It is a self-administered questionnaire, and responses are recorded on a 4-point Likert scale: 0 = never, 1 = it happened once or twice, 2 = it happened 3 to 5 times, and 3 = it happened 6 or more times. The global factorial structure showed good reliability fit (α = .67 for fathers and α = .71 for mothers). Following the criteria of Calvete (54), this questionnaire provides two total scores (psychological and physical) for each parent and a procedure for identifying cases as serious or not which is described in the results section.

In addition, an *ad hoc* open-ended question was added for this study asking young people to write about what they did immediately after these situations (“*What do you do after*?”). This question was only answered if the adolescent had engaged in at least one CPV behavior in the past year.

### 2.3 Procedure

This study is part of a larger research project in Spain (2022–2025) (12). The sample selection of adolescents followed a multi-level sampling approach: 1) simple random sampling by region, selecting 50% of the total regions of Spain; 2) non-probability convenience sampling, choosing provinces with the capital city of the selected autonomous community from the previous step; 3) stratified random sampling by type of institution (private, semi-private, or public) and area (urban or rural) in secondary or career-related education centers. Specifically, three educational centers per province were included (40% rural, 60% urban; 30% private-semi-private and 70% public); 4) purposive sampling was used to select classrooms within schools that agreed to participate in the research (e.g., letter A classrooms of each grade or based on availability on the day of the visit). Additionally, convenience sampling was employed to obtain a representative sample size and enough schools from the central-southern region of Spain. Six schools that had collaborated in previous research in the Madrid and Andalusia areas were invited to participate.

After obtaining approval from the ethics committee of the corresponding university (name of the university), high schools were contacted to request participation in the study. Once school administrations provided consent, parents or caregivers of the adolescents were informed about the study’s details and asked to provide consent for their children’s participation. Following parental consent, adolescents were given information tailored to their comprehension level to ensure a clear understanding of the study’s objectives and their right to withdraw at any time. Depending on their age, adolescents either signed an assent form (under 16 years) or a consent form (16 years and older). The assessment sessions, conducted in classrooms, lasted approximately 45 minutes.

### 2.4 Data and qualitative analysis

Prior to the qualitative analyses, descriptive analyses were conducted for sociodemographic (sex, age, or area) variables as well as for the total scores of the CPAQ-R instrument (53). Concerning CPAQ-R instrument, mean scores and standard deviations were calculated for general CPV scores—both psychological and physical—regarding fathers and mothers. Following the instrument’s guidelines, mild cases of CPV and severe cases of repeated behaviors were identified. Mild CPV refers to cases in which adolescents have engaged in at least one violent behavior toward their father or mother in the past year but do not meet the CPAQ criteria to be classified as severe CPV (54).

Using thematic analysis (55, 56), the main emerging themes in adolescents’ responses were identified. Only responses from adolescents who scored on any scale of the CPAQ-R (53) were coded. First, a code list was generated by one researcher, totaling 527 codes. Each response was categorized under one or more codes depending on whether the participant referenced a single or multiple content areas. Once the code list was finalized, researcher triangulation was conducted through code consultation with two researchers. After reaching a consensus on the codes, two independent researchers grouped the codes into various themes. A total of nine themes and 19 sub-themes were identified, with an inter-rater agreement rate on seven out of the nine themes and 18 out of the 19 sub-themes. Discrepancies were resolved by consulting a third researcher with expertise in CPV. The final classification included six themes and 17 sub-themes. Finally, the two independent researchers re-grouped all participant responses into the established themes, achieving 85% agreement, which led to the redefinition of two sub-themes. This process was conducted for both mild and severe cases of CPV. In the final phase of thematic analysis, each theme and sub-theme was defined (55). The themes and sub-themes presented in the results correspond to meaningful content regardless of the frequency of occurrence, thus following thematic analysis recommendations (56). Finally, as some adolescents mentioned several themes and sub-themes in their responses, a relational map was developed among sub-themes to understand the sequential process of adolescent responses following their CPV behaviors.

## 3 Results

### 3.1 CPV behaviors. frequency and severity of cases

A total of 410 adolescents reported engaging in at least one violent behavior in the past year (38.42% of the total sample of participants in the study, *n* = 1067). Specifically, 405 adolescents reported CPV behaviors directed toward their mother, and 389 toward their father. **Table 1** presents the descriptive results for the mean scores obtained by adolescents in both the psychological and physical dimensions of CPV, distinguishing between violence directed toward the mother and the father. Descriptive results indicated a higher average frequency of psychological CPV behaviors in cases involving both mothers and fathers.

**Table 1:** CPV Behavior: descriptive and severe cases.

	CPV Psychological Likert scale 0-3		CPV Physics Likert scale 0-3	
*M (SD)*	0.50 (0.39)		0.08 (0.19)	
	Mother (*n* = 405)	Father (*n* = 389)	Mother (*n* = 405)	Father (*n* = 389)
**Min-Max**	0-2.75	0-2.50	0-2.4	0-2
***M* (*SD*)**	0.56 (0.47)	0.44 (0.41)	0.09 (0.25)	0.07 (0.22)
**No severe cases** ***Fr* (%valid)**	379 (93.58%)	368 (94.60%)	378 (93.34%)	374 (96.14%)
**Severe cases** ***Fr* (*%*valid)**	26 (6.42%)	21 (5.39%)	27 (6.66%)	15 (3.86%)

The consideration of severe or non-severe has been taken in accordance with the CPAQ Authors' correction guidelines (54). 0 (never); 1 (it has happened once or twice); 2 (it has happened between three and five times); and 3 (it has happened six or more times).

Following the procedure outlined by the authors of the CPAQ (54), psychological CPV was categorized as severe or reiterative if at least one of the four behaviors occurred “more than six times in the last year” (option 3). For identifying cases of severe physical CPV, at least one of the five behaviors from this subscale had to be reported as occurring “3-5 times in the last year” (options 2 and 3). The results are presented in **Table 1**, showing frequencies and percentages relative to the total.

Regarding severe psychological CPV, adolescents reported recurrent CPV behaviors directed towards 6.42% of mothers (*n* = 26) and 5.39% of fathers (*n* = 21). In terms of physical violence, 6.66% of cases (*n* = 27) involved severe physical CPV behaviors towards mothers, while 3.86% were directed towards fathers (*n* = 15). Finally, a combined analysis of severity levels revealed that 88.54% of the adolescent sample did not exhibit severe CPV behaviors —either psychological or physical—towards either parental figure (mild cases *n* = 363), while 11.46% did report such behaviors towards one of their parental figures (severe cases *n* = 47). These 47 adolescents represent 4.40% of the total sample (*n* = 1067).

### 3.2 Adolescents’ responses After CPV in mild cases

A total of 363 responses were categorized from the question “What did you do after?” The codes were grouped into a total of six themes and 17 subthemes (see in **Table 2**). The following table provides a summary of the various themes and subthemes.

**Table 2:** Themes and subthemes of the different responses of the adolescent after mild CPV.

Themes	Subthemes	Definitions
**Individual emotional responses**	Feeling bad	Refers to responses in which the adolescent revealed that they cried or expressed discomfort, guilt, frustration, or sadness after engaging in CPV behavior.
	Self-calming	Refers to responses in which adolescents verbalized attempts to relax and reduce their arousal or tension levels associated with the conflict situation.
**Individual cognitive responses**	Remorse	Refers to thoughts about not having acted appropriately, the realization that their parents did not deserve such treatment, and ultimately the regret for their behavior.
	Reflection	Refers to responses in which adolescents paused to analyze their actions, consider the reasons behind them, and think about what to do after engaging in CPV behavior.
**Place safe**	Adolescent’s Room	This subtheme occurs when the adolescent retreats to their room.
	Significant non-cohabiting adults	This subtheme occurs when the adolescent leaves home and seeks support from other significant adults, such as a grandmother.
	Street	This subtheme occurs when the adolescent leaves home and goes out to the street without necessarily meeting friends or other significant adults.
**Activity safe**	Diverse activities	Refers to responses in which the adolescent stated that they engaged in an activity to distract themselves from the CPV situation, such as playing video games.
	Sleep	Refers to instances in which the adolescent retreats to their room to sleep.
**Response with father/mother**	Apologizing	This subtheme refers to responses in which adolescents initiate an interaction with their parents to apologize or ask for forgiveness, either immediately after the CPV behavior or after some time has passed.
	Hugging	Refers to the occurrence of a hug between the adolescent and the parent following the CPV situation.
	Resolving the problem	Refers to situations involving dialogue, communication, and discussions about what happened and the reasons behind it between the adolescent and their parent.
	Commitment to not repeating the violent behavior	This subtheme refers to responses from adolescents who explicitly stated that they communicated to their parents their intention not to act violently toward them again and expressed a firm decision to avoid such behavior in the future.
**Denial, avoidance, or reinforcement of violent behavior**	Not talking about behavior	Refers to instances when the adolescent indicated that the CPV situation was not discussed at home afterward, despite being aware of the severity of the situation.
	Normalizing the situation	Refers to instances when adolescents indicated that their behavior was not severe or when they allowed time to pass to normalize what had happened with their parents.
	Joking	Refers to instances when the adolescent makes jokes about the situation, downplaying the violent behavior to reduce tension.
	Justifying violent behavior	Refers to cases in which the adolescent includes in their responses a justification for their actions, attributing them to a prior behavior by their parent.

#### 3.2.1 Individual emotional responses

Adolescents reported experiencing various emotional responses following CPV behaviors. Specifically, they mentioned that after engaging in CPV, they felt bad and attempted to calm themselves to reduce their anger.

Feeling bad. This subtheme was the most frequently reported emotional response. 19% of adolescents identified feeling discomfort after the CPV incident, along with feelings of frustration and guilt. 69.4% of these responses were provided by girls. It was also common for adolescents to cry after engaging in CPV. This subtheme categorizes the responses of adolescents who explicitly mentioned feeling bad, feeling guilty, feeling frustrated, and crying. It is possible that the percentage of these feelings is higher, as other themes, such as remorse or apologizing, may carry an associated sense of discomfort even if adolescents did not mention it explicitly. An example statement is presented below:

“I felt very bad because it does not deserve, and I will apologize” (Subtheme: feeling bad. Girl. 18 years old).

Self-calming. 12.12% of adolescents reported attempting to calm themselves following a violent behavior episode. 68.7% of these responses were mentioned by girls. These adolescents employed various self-calming strategies, such as isolating themselves in their room, engaging in alternative activities like playing video games, going to sleep, going outside, or simply letting time pass. The responses used to calculate the percentage included only those in which adolescents explicitly mentioned the words ‘calm down,’ ‘relaxing,’ or ‘reduce anger,’ but not those in which they engaged in activities or went to their room without explicitly stating it was to calm down. Therefore, the percentage of adolescents employing self-calming strategies could be higher. An illustrative quote follows:

“I go to my room to calm down after the discussion” (Subtheme: self-calming. Girl, 18 years old).

#### 3.2.2 Individual cognitive responses

Adolescents also identified various reflective and cognitive responses following instances of CPV. The two subthemes that emerged were remorse and reflection.

Remorse. The response of remorse was expressed by the adolescents. In total, 9.37% mentioned feeling regret after engaging in adolescent-to-parent violent. 52.9% of these responses were provided by girls. This subtheme includes responses in which adolescents explicitly stated that they felt remorse. This feeling of remorse may or may not have been accompanied by an apology. An example is provided below:

“Regretting what I have done because you can’t do that to parents; they have given us everything and helped us in every way, and they don’t deserve that.” (Subtheme: remorse. Boy, 13 years old).

Reflection. This subtheme captures responses from adolescents who reported engaging in reflection after committing an act of CPV. Some adolescents mentioned recognizing the seriousness of their actions once they had calmed down. 4.68% of adolescents stated that they thought about their actions and considered ways to resolve the situation. Some adolescents mentioned needing time for reflection. 60% of these responses were mentioned by boys. An example is provided below:

“After doing it, I stop to think that it’s wrong and I apologize” (Subtheme: reflection. Girl, 14 years old).

#### 3.2.3 Place safe

We highlight those adolescents disclosed in their responses the safe places they chose to go after engaging in CPV. Adolescents frequently reported feeling the need to leave the setting where the CPV behavior occurred. They sought a different environment, away from the scene of violence, to calm themselves, cry, reflect, or speak with other significant adults. The following sections outline the most frequently mentioned subthemes.

Adolescent’s Room. 11.29% of adolescents explicitly mentioned going to their room, usually with the purpose of being alone, calming down, crying, or reflecting. 53.1% of these responses were provided by girls. The percentage of adolescents who went to their room may be higher; however, this percentage only includes those who explicitly stated in their responses that they went to their room. An example response follows:

“*I lock myself in my room until the anger passes*” (Subtheme: adolescent’s room. Girl, 16 years old).

Significant non-cohabiting adults. Three girls mentioned that they turned to non-cohabiting, significant adults for support, conversation, and to help calm themselves after engaging in CPV behavior. These significant adults included the other parents, a sister, or a grandmother. An example of this response is:

“*I usually leave and go clear my mind at my grandmother’s house*” (Subtheme: significant non-cohabiting adults. Girl, 13 years old).

Street. 2.20% of adolescents mentioned that they needed to go outside or to the street to calm down and to reduce the tension at home. 66.6% of these responses were provided by girls. An example is provided below:

“I go out into the street” (Subtheme: street. Boy, 18 years old).

#### 3.2.4 Activity safe

Adolescents disclosed various activities they engaged in after the CPV behavior. These activities may serve as a strategy to avoid conflict, to calm down, to stop thinking about the incident, or simply to stay distracted. These activities can have an active component, such as playing video games, or a passive one, such as sleeping.

Diverse activities. 1.38% of adolescents mentioned that after engaging in CPV behavior, they would engage in activities such as playing video games, watching movies, or listening to music. We found the same number of boys and girls reporting this subtheme. Additionally, these activities are typically done alone with the aim of escaping from the conflict and calming themselves. An example is provided below.

“*I lock myself in my room and listen to music*” (Subtheme: diverse activities. Girl, 13 years old).

Sleep. 1.38% of adolescents mentioned that they went to sleep in their room. 75% of these responses were provided by boys. Sleeping can be interpreted as an avoidance behavior in response to conflict, but also as a strategy for self-calming. An example response is:

“ *Sleeping to forget about things*” (Subtheme: sleep. Boy, 12 years old).

#### 3.2.5 Responses with the father or mother

This theme was the most common among adolescents. In this theme, we grouped responses where adolescents reported engaging in actions involving their parents. Specifically, responses included actions such as apologizing, hugging their mother or father, explaining the reasons for their behavior, resolving the issue, or committing not to repeat the behavior. These relational actions with the victim of the violent behavior may reflect adolescents’ expressions of remorse or efforts to repair the harm done. The following subthemes are identified.

Apologizing. It was the most frequent response among adolescents. This behavior was associated with the adolescent’s remorse, acknowledgment of inappropriate behavior, or recognition of harm caused. This subtheme includes responses where adolescents explicitly stated they had apologized; specifically, 70.52% of adolescents mentioned this behavior. 59.2% of these responses were provided by girls. However, this percentage could be higher, as many adolescents expressed regret without elaborating further or mentioned other reparative behaviors without explicitly using the term “apology.” Some adolescents combined apologies with other emotional and/or cognitive responses, such as calming down, or with additional actions involving their parent, like giving a hug. An example follows:

“*After being more calm to apologize*” (Subtheme: apologizing. Boy. 17 years old).

Hugging. Some adolescents (3.58%) reported hugging their mother or father following a CPV incident. 61.5% of these responses were mentioned by girls. This gesture was typically preceded by feelings of remorse and an apology. An illustrative example is:

“*I apologized and hugged her*” (Subtheme: hugging. Girl, 13 years old).

Resolving the problem. Some adolescents (5.78%) indicated that they took steps to resolve the issue that led to the CPV behavior. This subtheme emerged in the same number of boys and girls. Communication and dialogue were frequently employed by these adolescents to discuss the situation with their parents, explain the reasons for their behavior, and to try to resolve the problem with them. An example response is:

“*We make peace and reach an agreement together”* (Subtheme: resolving the problem. Boy, 18 years old).

Commitment to not repeating the behavior. 1.93% of the adolescents apologized, committing not to repeat the violent behavior. 71.4% of these responses were mentioned by girls. An illustrative example is:

“*I apologized and swore I would never do it again*” (Subtheme: commitment to not repeating the behavior. Boy, 12 years old).

#### 3.2.6 Denial, avoidance, or reinforcement of violent behavior

A substantial group of adolescents did not mention any emotional response or impact following incidents of CPV, nor did they engage in apologizing or reparative behaviors with their parents. This theme includes adolescents who chose not to discuss the situation, those who normalized the behavior, those who joked about their actions, and those who justified their violent behavior. The following four subthemes are identified.

Not talking about behavior. Some adolescents acknowledged that they had not spoken to their parents about the violent behavior they had exhibited and did not take any action regarding the incident. Specifically, 3.03% of adolescents reported this response. 54.5% of these responses were mentioned by girls. An illustrative example is:

“*Nothing, I just let it go*” (Subtheme: not discussing the situation. Girl, 16 years old).

Normalizing the situation. In certain cases (2.75%), adolescents indicated that they continued to interact with their parents as if nothing had happened, effectively avoiding acknowledgment of violent behavior. 66.6% of these responses were mentioned by girls. An example response follows:

“*Pretending nothing happened*” (Subtheme: normalizing the situation. Girl, 18 years old).

Joking. Two girls and one boy reported joking with their parents about the violent behavior, often in situations where the violent behavior involved verbal insults. These adolescents generally told their parents it was a joke as a strategy to reduce tension. An example response is:

“Telling them it was a joke, to ease the situation” (Subtheme: joking. Girl, 15 years old).

Justifying violent behavior. 2.20% of adolescents justified their violent behavior as a response to their parents’ actions, and some even mentioned feeling no remorse or expressed satisfaction with their actions. 52.3% of these responses were mentioned by girls. An example of this subtheme is provided below:

“Telling them that these things wouldn’t happen if they were fairer with me” (Subtheme: Justifying violent behavior. (Boy, 17 years old).

### 3.3 Relationship between adolescents’ responses after CPV in mild cases

When adolescents were asked what they do after engaging in CPV behaviors, they often described several actions in a sequence, following the structure “first [ … ] and then [ … ]”. Consequently, subthemes were sometimes interconnected. The most frequent subtheme, “apologizing,” was the one most commonly linked to other subthemes. In some cases, subthemes occurred before apologizing “first [ … ] and then I apologize”, while in others, subthemes appeared after apologizing “first I apologize and then [ … ]”. Therefore, based on the occurrence of these patterns, we propose a model that emerges from the relationships between the subthemes, with apologizing as the central component. In this model, we identify three phases: Phase 1 includes the subthemes that appear in responses before apologizing, Phase 2 consists of the act of apologizing itself, and Phase 3 comprises the subthemes that occur after apologizing. We propose the following figure, which illustrates the potential phases that an adolescent may go through after exhibiting CPV.

We present below the percentage of adolescents who provided a single response within a subtheme and the phase in which it would be situated within the model, as well as the percentage of adolescents who provided a response describing multiple behaviors following CPV and the phases in which those behaviors would be situated.

Adolescents with a single response corresponding to a subtheme in Phase 1 (21.49%). These adolescents only referred to individual responses following CPV, which may be emotional or cognitive in nature and may also involve seeking a safe place or engaging in other safe activities after the violent incident. This group includes adolescents who mentioned feeling bad after violent behavior, attempting to calm themselves, experiencing remorse, reflecting on their actions, and going to safe spaces like their room, among other options. However, this group does not include those who, in addition to engaging in these behaviors, explicitly mentioned apologizing to their parents.

“Trying to calm myself down” (Boy, 19 years old).

Adolescents with a single response corresponding to the subtheme “apologizing” in Phase 2 (36.91%). This group was the largest. These adolescents only explicitly stated that, following the CPV behavior, they apologized to their parents. They did not mention any individual responses characteristic of Phase 1. However, although the adolescents explicitly identified apology as their only response to the CPV situation, it is likely that they experienced some of the emotional or cognitive responses characteristic of Phase 1 before reaching the point of apology, even if they did not make this explicit.

“Asking for forgiveness (from my father or mother)” (A common response among multiple adolescents).

Adolescents with a single response corresponding to a subtheme in Phase 3 (1.65%). In these cases, we refer to adolescents who engage in behaviors with their parents other than apologizing. This group includes those who explicitly mentioned that they talked with their parents to resolve the issue, committed that it would never happen again, or gave a hug as a reparative action. Although these adolescents do not explicitly refer to the responses from Phase 1 and Phase 2, it is possible that these phases occurred prior to these behaviors, even if they were not mentioned in their responses.

“Talking about what happened” (Girl, 16 years old).

Adolescents with multiple responses corresponding to subthemes in Phase 1 preceding the apology in Phase 2 (24.24%). This was the second largest group. These adolescents explicitly mentioned that they first felt bad, experienced remorse, or needed to calm down before proceeding to apologize.

“Feeling bad, crying, thinking, and then apologizing” (Girl, 16 years old).

Adolescents who first apologized in Phase 2 and then engaged in behaviors corresponding to subthemes in Phase 3 (8.26%). These adolescents expressed that they apologized to their father and mother and additionally talked with them about the exhibited behavior or the problematic situation. Therefore, these adolescents did not limit themselves to merely apologizing; instead, they took a more dialogue-based approach, including physical gestures such as hugging or committing not to repeat the violent behavior.

“Apologizing and calmly talking things over” (Boy, 16 years old).

**Figure 1:**
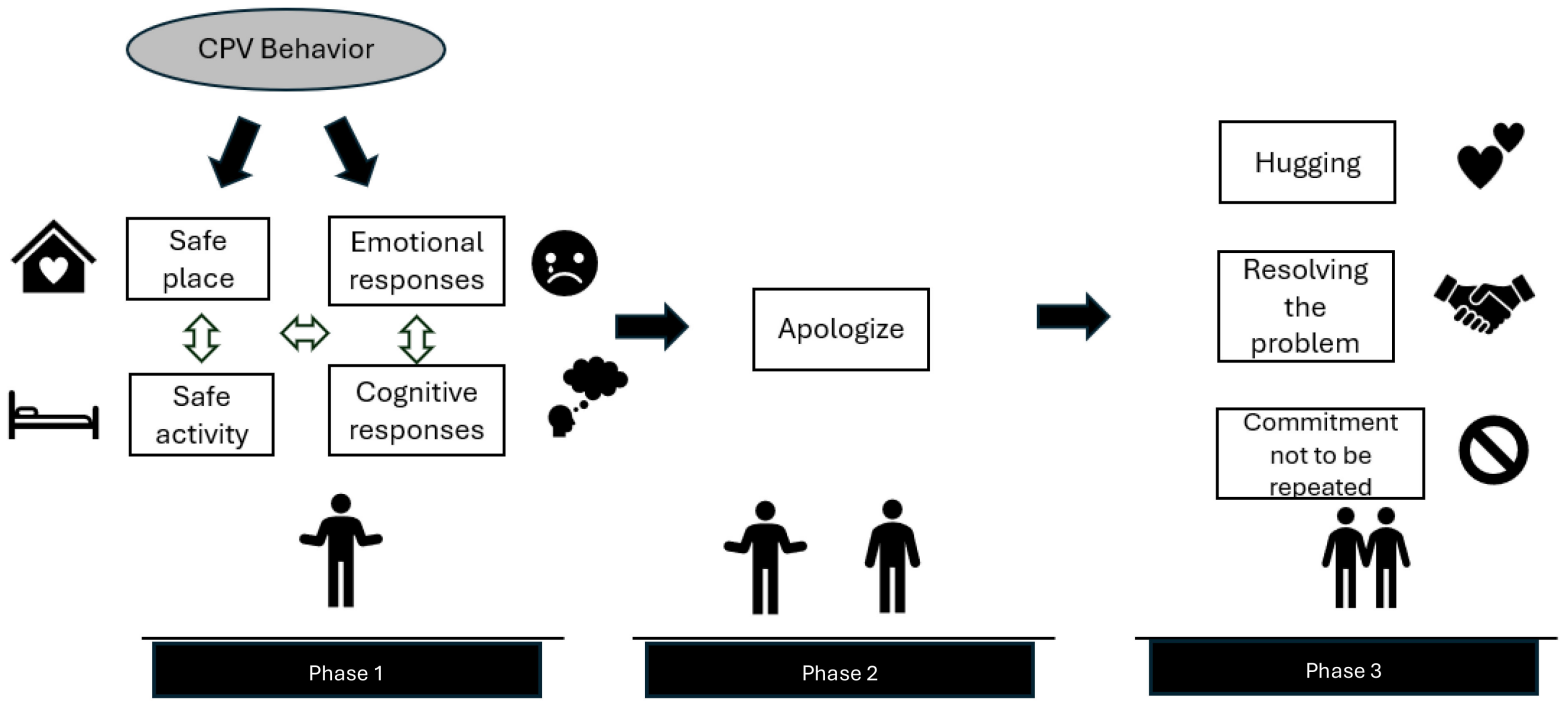
Adolescents' Responses to CPV: Sequential Subtheme Relations.

Adolescents who responded with multiple behaviors corresponding to the subthemes in Phase 1, then apologized in Phase 2, and subsequently engaged in behaviors corresponding to the subthemes in Phase 3 (3.03%). These adolescents explicitly mentioned in their responses the entire process outlined in **Figure 1**. That is, they described how the violent behavior initially had an emotional impact on them, elicited a cognitive response, or required them to seek a safe space to calm down before apologizing. Furthermore, after apologizing, they talked with their father or mother about the conflict, reaching agreements or demonstrating reparative behaviors.

“Once my anger had passed, I felt remorse, apologized, and even gave them a hug and a kiss” (Girl, 16 years old).

In this sequential process of responses that adolescents engage in after violent behavior (**Figure 1**), those adolescents who normalize violent behavior or justify, deny, or downplay their actions were not included. Altogether, this group comprised 8% of the total adolescents in mild CPV. In summary, while most adolescents respond to their violent behavior, some others normalize these actions, justify them, joke about them, or avoid discussing them.

### 3.4 Adolescents´ responses after CPV in severe cases

In cases of severe CPV (*n* = 47), most subthemes from mild CPV emerged, except for the subthemes of sleeping, committing to not repeat the behavior, or joking about the violent behavior. Additionally, a new theme emerged, which, although reported by only one girl, is of particular relevance: risk behaviors such as self-harm. Like mild CPV, apologizing to one’s father or mother remained the most frequent subtheme among adolescents with severe CPV. However, unlike mild CPV, the second most frequent subtheme was not talking about the behavior, followed by normalizing the violent situation. In fact, not talking about the violent situation and normalizing it were the subthemes that showed the greatest increase in percentage in severe CPV relative to mild CPV. These two subthemes, along with justifying their violent behaviors, account for a total of 23.4% of adolescents with severe CPV, while 8% of adolescents displayed these subthemes in mild CPV. In contrast, the three subthemes that showed the greatest decrease in percentage relative to mild CPV were apologizing, feeling bad, and attempting to calm down. **Table 3** presents the percentage of subtheme occurrences in severe CPV, along with the percentages for mild CPV, to illustrate the differences between the two.

**Table 3:** Percentage of themes and subthemes in adolescents with mild and severe CPV.

Themes	Subthemes	% Mild CPV	% Severe CPV
**Individual emotional responses**	Feeling bad	19	6.38
	Self-calming	12.12	6.38
**Individual cognitive responses**	Remorse	9.37	6.38
	Reflection	4.68	6.38
**Place safe**	Adolescent’s Room	11.29	6.38
	Significant non-cohabiting adults	0.008	2.13
	Street	2.20	4.25
**Activity safe**	Diverse activities	1.38	4.25
	Sleep	1.38	0
**Behavior at risk**	Self-harm	0	2.13
**Response with father/mother**	Apologizing	70.52	40.42
	Hugging	3.58	2.13
	Resolving the problem	5.78	6.38
	Commitment to not repeating the violent behavior	1.93	0
**Denial, avoidance, or reinforcement of violent behavior**	Not talking about behavior	3.03	10.64
	Normalizing the situation	2.75	8.51
	Joking	0.008	0
	Justifying violent behavior	2.20	4.25

% Mild CPV: 1 (it has happened once or twice) or 2 (it has happened between three and five times). % Severe CPV: 3 (it has happened six or more times).

## 4 Discussion

In the last decade, research focused on understanding CPV from an ecological-systemic approach has increased (57). These studies have primarily aimed at identifying the factors involved in CPV using quantitative methods, often disregarding the narratives of those directly involved in these situations (52). Furthermore, the focus has mainly been on the antecedents of CPV, while significant gaps remain regarding what occurs following CPV behavior. This study makes an important contribution to understanding CPV by giving voice to adolescents who engage in violent behavior toward one of their parents and focusing on what happens after the violent incident. Understanding how adolescents act after engaging in violent behaviors can contribute to improving treatments by identifying emotions, cognitions, or reparative behaviors that the adolescent may exhibit.

In this study, 38.42% of adolescents (n = 410 out of 1067) reported engaging in at least one violent behavior toward their parents in the past year, underscoring the prevalence of CPV, while 4.40% (*n* = 47) exhibited severe CPV. The prevalence of recurrent CPV behaviors was lower than that reported by Calvete et al. (58). Consistent with previous research, psychological CPV was more common than physical CPV, with both fathers and mothers as targets (59). Nonetheless, the fact that one in twenty adolescents displays severe and recurrent violent behavior in a random sample from Spanish high schools is particularly concerning.

Recurrent and severe violent behaviors were more frequently directed at mothers than fathers, particularly in cases of physical violence, aligning with previous studies examining the role of the victim’s gender (9). These findings underscore the importance of identifying CPV behaviors early, as evidence suggests they often escalate gradually, increasing in both intensity and frequency. Early detection, coupled with a deeper understanding of the dynamics underlying family conflicts, is essential to preventing escalation. Such efforts are critical for providing timely support to adolescents and their families, promoting effective prevention strategies, and mitigating the long-term impact of CPV (37).

In response to the question, “What do adolescents do after assaulting their father or mother?” The 363 responses from adolescents in mild cases were categorized into six themes and 17 subthemes. The most frequent individual responses were apologizing to the parent, feeling bad, trying to calm down, and going to their room. However, a wide range of responses emerged, which we categorized into individual responses of an emotional nature (with “feeling bad” being the most frequent) or cognitive nature (with “remorse” being the most frequent); activities (ranging from listening to music to sleeping); a safe place to go after the risk behavior (the room was identified as the safest place); behaviors involving interaction with the parent (with “apologizing” being the most frequent); or denial, avoidance, or reinforcement of violent behavior, with the most frequent response being to avoid talking to the parent about the violent behavior. These responses were not mutually exclusive, with it being common to find both individual (emotional, cognitive, and behavioral) and interpersonal (interaction with the parent) responses in the same adolescent. According to Bonanno et al. (41), strategies for coping with stressful situations are not inherently suitable or unsuitable; they depend on regulatory flexibility (the characteristics of the context, the repertoire of available strategies, and feedback on the effectiveness of the strategy applied). For this reason, it is considered that a broad repertoire of responses in adolescents may increase the likelihood of better adapting to each conflict situation.

This study identified two groups of adolescents: one group that acknowledged a significant impact of CPV and expressed either individual responses that reflected this impact or relational responses aimed at resolving the conflict with their parent (Group 1: 92%); and another group that did not acknowledge CPV, instead normalizing and justifying it (Group 2: 8% of the adolescents). This group was more frequent (23.40%) among adolescents exhibiting severe and recurrent CPV. These adolescents (those with severe and recurrent CPV) appear to lack awareness of the severity of their behaviors, ignoring and justifying their repeated violent actions, in contrast to those with mild CPV who seem to respond more responsibly to their violent behaviors.

### 4.1 Adolescents who acknowledge the impact of CPV: a three-phase model

Focusing on the first group, **Figure 1** presents the sequential responses representing most adolescents in Group 1, who, after engaging in violent behavior, feel bad, try to calm down, seek a safe place, and initiate a process aimed at reflection, remorse, acknowledgment, and repair of the harm caused, ranging from apologizing to discussing the problem with the parent.

Specifically, after engaging in violent behavior toward their father or mother, adolescents exhibited a wide range of individual responses—emotional, cognitive, and/or behavioral (Phase 1). In this initial phase, adolescents responded emotionally (feeling bad and self-calming), cognitively (remorse and reflection), behaviorally by seeking a safe place (such as their room, a non-cohabiting significant adult’s place, or the street), and/or by engaging in distracting activities (various activities such as listening to music or sleeping). Analysis of the emotional responses reveals the adolescent’s distress after committing the violent act, with expressions of frustration, guilt, and crying over what happened. These reactions may explain the presence of self-harming behaviors in managing negative emotional states and avoiding anxiety, as observed exceptionally in one adolescent (60). However, most adolescents reported needing self-calming after the violent act. These responses support the hypothesis that adolescents experience distress and need to calm down, thus engaging in emotional regulation strategies (48). This phase also includes behavioral responses that help the adolescent reduce emotional intensity (48). For instance, leaving the setting where the CPV behavior occurred to disconnect or seek support from others elsewhere (60, 61). These behavioral responses (seeking a safe place) along with engaging in an activity (various activities or sleep) have proven to be supportive needs for adolescents involved in intrafamily violence situations (62). In this phase, the combined presence of these responses is considered beneficial as it invites adolescents to reduce the emotional tension experienced (e.g., crying, going to a safe place, typically their room) and to become aware of the consequences of their actions (e.g., realizing and reflecting on what happened). Cognitive responses are key in this process as they allow adolescents to gain awareness of the issue and contribute to the assumption of responsibility. In contrast to cases where aggression escalates, the adolescent’s expression of remorse, finding a safe environment, and/or seeking support from a trusted adult facilitates calming down, reflecting on the conflict, and taking steps toward apologizing with a sense of responsibility for their actions (63). In the responses from Phase 1, distinct behaviors between boys and girls can be observed in some subthemes. One of the largest differences in percentages was that girls reported feeling bad and attempting to calm down after CPV behavior more often than boys. This is consistent with previous studies on CPV and gender, which show that girls tend to exhibit greater emotional attention and empathy than boys (46). Conversely, another gender difference found was that boys were more likely than girls to go to sleep as a way to avoid the emotions associated with the conflict, while more girls, in line with previous studies on coping strategies in stressful situations (44, 45) reported seeking support from others, in this case from other significant adults—a subtheme that barely appeared among boys.

The responses in Phase 1 are essential for adolescents to progress in the process of repairing the harm caused. Phase 2 (apologizing) is fundamental for this progression and is closely linked to Phase 1. Apologizing was the subtheme most frequently mentioned by adolescents. It is necessary for adolescents to reach Phase 2 after a period of reflection or moratorium on the incident, during which they have managed to reduce their level of emotional intensity. Phase 2 refers to the moment of apologizing to the abused parent. Therefore, this act of apologizing implies accepting responsibility for the violent action. However, it would be interesting to understand the reasons why 70.52% of adolescents in mild CPV (and the 40.42 in severe CPV) apologize to their parents—that is, whether adolescents do so from recognizing the harm caused by their actions, due to an inability to manage their own negative emotions, out of fear of their parents’ response, or from a combination of various reasons. Currently, it is known that acknowledgment of harm is a key variable in psychotherapeutic interventions with aggressors, facilitating reparative processes and reducing the likelihood of recurrent violent behavior (64, 65).

Finally, we found that approximately 10% of adolescents, in addition to apologizing, engage in behaviors such as hugging their parents, resolving the problem through dialogue, or committing to not repeat the violent behavior (Phase 3). Among these three subthemes, problem resolution through dialogue was the most frequent. Although there is no single effective strategy for adolescents to manage family conflicts, focusing on problem resolution has proven useful for boys and girls at this age (66). It is known that greater use of adaptive coping strategies is associated with fewer externalizing problems (including violent behaviors) as well as internalizing problems (67). Therefore, completing the third phase not only benefits the parent-child relationship but also supports the adolescent’s personal well-being.

### 4.2 Adolescents who normalize, ignore, joke about, and justify CPV

In contrast to the previous group of adolescents, 8% of adolescents who engage in occasional violent behavior and 23.40% of adolescents with severe and recurrent CPV normalize, ignore, joke about, and justify their actions. This could be due to various factors: (a) the violent behavior has been normalized due to a history of family violence (11, 68, 69); (b) they do not recognize their actions as abusive, especially in cases of financial abuse, humiliation, intimidation, or physical aggression without injury (30); (c) the relational control dynamic continues (28, 70); (d) they lack the capacity to manage or resolve the situation (70) and this may be comparable to the psychological withdrawal experienced by victims of gender-based violence (71). The combination of these reasons could predict an increase in CPV behaviors in this second group of adolescents. These adolescents seem to lack awareness of the problem, as well as remorse or a commitment to change. Additionally, adolescents with severe CPV are less likely to report feeling bad compared to those with sporadic violent behaviors. Some authors have reported emotional difficulties in adolescents with violent behaviors (28); thus, it may be that in cases of recurrent CPV, the adolescent has normalized their behavior and does not experience an emotional impact, or that they are unable to recognize the severity of their actions.

Previous studies report a profile of adolescents who engage in CPV characterized by high levels of anger, emotional instability, impulsivity, low cognitive flexibility, erroneous attributions, a hostile perception of parental authority, and substance use (33, 72). The presence of any of these characteristics in an adolescent would clearly hinder the implementation of adaptive cognitive-emotional regulation strategies that encourage reflection (e.g., acceptance, perspective-taking, or positive reappraisal) and would instead facilitate maladaptive strategies (e.g., rumination, blaming others). For instance, it is known that uncontrolled anger impairs the ability to think rationally (73) and that rumination seems to increase adolescent aggressiveness (73, 74). These factors could explain why adolescents might struggle to calm down, as observed in Group 1, and instead may attempt to normalize their behavior.

## 5 Practical implications

The findings of this study provide essential insights into the post-CPV behaviors of adolescents, offering significant implications for intervention strategies aimed at addressing CPV and supporting affected families. Understanding the sequential emotional, cognitive, and behavioral responses that occur after a violent episode—particularly in adolescents who acknowledge their behavior and attempt reparative actions—can enhance the effectiveness of therapeutic approaches.

Regarding Phase 1 of the model, this study identifies some key elements for preventive intervention in CPV: adolescents respond to their own violence through various actions (emotional, cognitive, and/or behavioral), with emotional suffering and gestures of apology prevailing. Emotional dysregulation immediately after violent behavior can lead to repetitive and similar responses in both parents and adolescents, reinforcing the cycle of violence (75). For this reason, therapeutic models could include structured activities like mindfulness exercises to help adolescents de-escalate emotional tension following an episode of CPV. This approach aligns with evidence suggesting that emotional regulation is a critical component of reducing maladaptive behaviors and fostering adaptive coping mechanisms (48, 63).

Among behavioral responses, adolescents’ needs for a safe space (in mild CPV cases, the room is recognized as a safe place, but not in severe CPV cases), talking calmly with someone about what happened, or engaging in distracting activities to help calm down are notable so it is essential restore the sense of security and work on empowerment and self-esteem as proposed in the Trauma-Informed approach (76) or the nonviolent resistance model because they prevent the escalation of violence (37, 77).

Regarding cognitive responses, the process of reflection or moratorium is central to recognizing the harm inflicted and subsequent acknowledgment. In this sense, working with adolescents should focus not only on the emotional and behavioral but also on the cognitive aspects. Social information processing theory (78, 79) emphasizes the importance of changing habitual biases in aggressive individuals, such as attributing hostile intentions, selectively attending to negative elements of the situation, setting revenge-oriented goals, positively evaluating the aggressive response, and engaging in aggressive behavior. Maintaining these metacognitive beliefs increases the likelihood of aggressive behavior, and aggressive behavior in turn exacerbates these biases, creating a vicious cycle that needs to be broken (80). Metacognition should be recognized as an intervention target, and it is advisable to address it considering the systemic nature of CPV (81). From this perspective, metacognition offers several benefits, as it enables working from a shared responsibility in a clear and balanced manner, enhances the achievement of individual metacognitive processes, facilitates the shift from typical hostile beliefs, and strengthens the development of supportive strategies among family members (81, 82), as is extensively described in the Educational and Therapeutic Treatment Program for Family Maltreatment (83) or the BreakChange program (84).

Phase 2, characterized by apologizing, highlights the importance of helping adolescents acknowledge the harm caused and take responsibility for their actions. Interventions could focus on promoting authentic expressions of remorse, rather than fear-driven or superficial apologies, to ensure meaningful relational repair. Cognitive-behavioral approaches could be employed to explore adolescents’ motivations for apologizing and reinforce their understanding of the impact of their behavior on family dynamics. By fostering genuine acknowledgment and appropriately managing blame, intervention programs can reduce the risk of recurrent CPV (64, 65), as such approaches have been shown to inhibit antisocial behavior (51).

The transition to Phase 3, where adolescents engage in dialogue, problem-solving, or commitments to behavioral change, highlights the value of teaching effective communication and conflict resolution skills. These behaviors not only benefit the parent-child relationship but also contribute to adolescents’ emotional well-being and long-term social competence. Structured family therapy sessions from a systemic approach (85–87) could be designed to guide families in collaboratively addressing conflict, encouraging open dialogue, and promoting mutual understanding. Therefore, working on communication and relationship patterns should be another key goal in CPV interventions to promote lasting changes in family dynamics (27).

For adolescents in Group 2, who normalize or justify their violent behavior, interventions must focus on addressing their lack of awareness and the emotional impact of their actions. These adolescents may require targeted efforts to disrupt maladaptive narratives and challenge normalized views of violence. Psychoeducational interventions could emphasize building empathy, cognitive flexibility, and perspective-taking skills to help them recognize the harm caused by their behavior. Additionally, therapeutic approaches might incorporate emotion-focused techniques to address underlying emotional instability, anger, or impulsivity that hinder their capacity for self-reflection and adaptive coping (73, 87).

Overall, the findings of this study suggest that tailored interventions targeting the post-CPV behavioral patterns identified in adolescents can help reduce recurrence, improve family dynamics, and support adolescents’ psychological and emotional growth. These insights highlight the need for comprehensive, multi-phase intervention programs that adapt to the specific needs of adolescents based on their responses to CPV incidents.

## 6 Limitations

This study presents several limitations that should be considered. First, by including only adolescent responses in the analysis, their perspectives may not align with those of their parents (88), which is crucial for understanding the different nuances of the problem. Additionally, we lack information on parental behavior and its effect on adolescents’ responses to CPV. For instance, in severe cases, Omer (37) suggests that an adolescent’s participation in an escalation of aggression also depends on the parent’s behavior during the conflict. Therefore, understanding parental responses would have helped clarify adolescent responses. Likewise, incorporating family characteristics such as parenting competencies would have contributed to a better understanding of adolescent responses. Second, due to the qualitative approach, we did not statistically compare differences based on age, gender, family structure, and other variables that may be related to CPV. It would be interesting for future studies to statistically examine whether there are differences in performance between boys and girls, between younger and older adolescents, and based on the severity of CPV (mild cases versus severe cases). Third, the adolescent responses were written; thus, using individual interviews could have allowed for deeper exploration of each response, the reasons for their violent behavior, and their parents’ actions.

## 7 Conclusions

This study makes an important contribution to understanding what adolescents do after engaging in violent behavior. A model is proposed for the adolescent’s response process, directing attention to emotional awareness, reflection, acknowledgment, and repair of the harm caused after violent behavior. Thematic analysis has provided insight into adolescents’ reality, listening to what they feel, think, and do after engaging in CPV. This study highlights the importance of preventive work on CPV, emphasizing the need to address the issue when the adolescent’s first violent behaviors toward a parent emerge to reduce the likelihood of violent escalation. A three-phase model organized around the most frequent response among adolescents who engage in CPV (apologizing to their parent) is shown. This three-phase model can help professionals identify the behaviors exhibited by adolescents after a violent episode and serves as a guide to achieving Phase 3, focused on dialogue, resolving the problematic situation, and committing not to repeat the violent behavior.

This study highlights the ongoing challenges related to CPV: in a community sample, nearly half of the adolescents displayed some form of violent behavior toward their parents, and one in ten met the criteria for severe and recurrent CPV, which is particularly concerning. Furthermore, among adolescents exhibiting severe violent behavior, 23.40% normalized, ignored, or justified their actions, a finding that raises significant concern. Therefore, given the rising cases of CPV, preventive measures such as family support programs and initiatives promoting positive adolescent development should continue to be developed and implemented.

We propose that future research evaluates the applicability of the model across diverse gender, cultural, socioeconomic, and clinical contexts, which could help refine its phases and adapt them to various settings. The model could also serve as a foundation for developing tools to measure adolescents’ emotional, cognitive, and behavioral responses following episodes of child-to-parent violence (CPV), facilitating assessment in therapeutic and research contexts. Longitudinal studies would be particularly valuable to examine how adolescents’ responses to CPV evolve over time and to explore the impact of specific interventions. Gender differences should also be considered in future studies. Additionally, we recommend further investigation into the role of dialogue and conflict resolution skills in facilitating the transition to Phase 3 and their influence on improving family dynamics and reducing CPV recurrence. Finally, given that a significant proportion of adolescents with severe violent behaviors normalize, justify, or ignore their actions, future studies could explore strategies to address these metacognitive beliefs and promote sustainable behavioral change.

In summary, this study shows practical implications for intervention with adolescents engaging in CPV, such as the importance of managing emotional intensity in the moment, identifying a safe place, having people or activities available to help them calm down, reflecting on their behaviors, acknowledging the harm caused, and providing them with space to apologize to their parents and discuss what happened, establishing agreements and reducing conflict.

## Data Availability

The original contributions presented in the study are included in the article/supplementary material. Further inquiries can be directed to the corresponding author.
